# Ghrelin agonist does not foster insulin resistance but improves cognition in an Alzheimer’s disease mouse model

**DOI:** 10.1038/srep11452

**Published:** 2015-06-19

**Authors:** Nicolas Kunath, Thomas van Groen, David B. Allison, Ashish Kumar, Monique Dozier-Sharpe, Inga Kadish

**Affiliations:** 1 Department of Cell, Developmental and Integrative Biology, University of Alabama at Birmingham, Birmingham AL, USA; 2 Office of Energetics; Nutrition Obesity Research Center; Department of Nutrition Sciences, University of Alabama at Birmingham, Birmingham, AL, USA; 3 Department of Clinical Research, Max-Planck-Institute of Psychiatry, Munich, Germany

## Abstract

The orexigenic hormone ghrelin, a potential antagonist of the insulin system, ensures sufficient serum glucose in times of fasting. In the race for new therapeutics for diabetes, one focus of study has been antagonizing the ghrelin system in order to improve glucose tolerance. We provide evidence for a differential role of a ghrelin agonist on glucose homeostasis in an Alzheimer’s disease mouse model fed a high–glycemic index diet as a constant challenge for glucose homeostasis. The ghrelin agonist impaired glucose tolerance immediately after administration but not in the long term. At the same time, the ghrelin agonist improved spatial learning in the mice, raised their activity levels, and reduced their body weight and fat mass. Immunoassay results showed a beneficial impact of long-term treatment on insulin signaling pathways in hippocampal tissue. The present results suggest that ghrelin might improve cognition in Alzheimer’s disease via a central nervous system mechanism involving insulin signaling.

Ever since the discovery of ghrelin as a ligand of the growth hormone secretagogue receptor in 1999^1^, our understanding of the versatile role of ghrelin in mammals has constantly expanded. The characterization of ghrelin has spanned its actions as an orexigenic hormone leading to weight gain and adiposity in rodents^2^,^3^, to the stimulation of appetite in humans^4^, its impacts on cognitive processes in rodents^5^,^6^ and humans^7^,^8^,^9^, and its role as a neuroprotective agent in neurodegenerative diseases^10^,^11^,^12^,^13^,^14^. Ghrelin’s involvement in glucose metabolism became apparent very early^15^,^16^, with evidence for a differential role of des-acyl ghrelin^17^,^18^. Recently, many groups have focused on the interactions of ghrelin with the insulin system in humans^9^,^19^. Antagonizing the insulinostatic ghrelin system has repeatedly been suggested as a novel mechanism by which to improve glucose homeostasis in humans. However, to our knowledge, none of the studies of the interactions of ghrelin with glucose homeostasis have addressed the long-term impact of ghrelin administration on a mammal.

Our group showed previously that administration of a ghrelin agonist leads to improved cognition and improved markers of pathology in an Alzheimer’s disease mouse model, even in the absence of caloric restriction^12^. The pathophysiological correlations between Alzheimer’s disease, impaired glucose metabolism, and diabetes are well established^20^,^21^,^22^, and elevated serum glucose levels have been shown to be an independent risk factor for dementia in humans^23^. In the present study, therefore, we aimed to investigate the long-term effects of a ghrelin agonist given for 4 months on Alzheimer’s disease pathology, cognition, and metabolism in the same mouse model fed a high–glycemic index (GI) diet as a constant challenge for glucose homeostasis. We hypothesized to see either (i) a detrimental effect of ghrelin agonist treatment in combination with this diet on cognitive and metabolic endpoints owing to interference with insulin signaling and consequently higher overall blood glucose levels or (ii) a protective effect as seen in our previous study via a thus far unknown mechanism.

## Results

### Ghrelin agonist acts as a long-term cognitive enhancer in spatial learning

Other groups have previously reported increased levels of anxiety in neonatal chicks and rats in the open field test after ghrelin administration^6^,^24^. In several preliminary tests we performed to exclude any a priori differences between groups, we did not observe any statistically significant differences between groups in categories such as anxiety or exploration activity (open field, zero maze, dark-light-box; see methods; data not shown). We also did not detect any significant group differences in performance in an object recognition task, which had been observed to be improved by short-term ghrelin treatment by another research group^25^.

**Figure 1:**
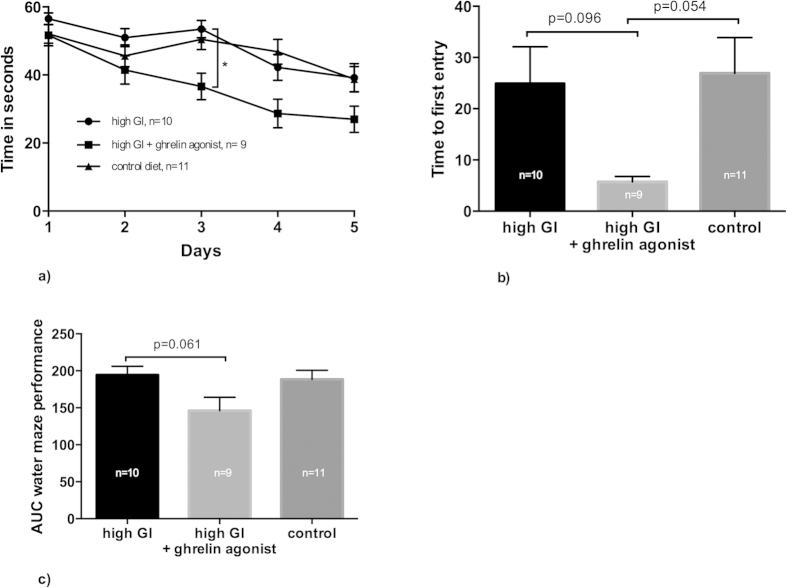
Ghrelin-agonist-treated animals performed better in a water maze test. They showed a faster learningcurve than did the group fed a high-GI diet alone. Intra-day differences between high-GI and high-GI + ghrelin agonist groups were significant for day 3 ((**a**), one-way ANOVA followed by post-hoc Tukey’s multiple comparisons test, p = 0.026), an Area-Under-The-Curve (AUC)-comparison for the graphs in (**a**) revealed that ghrelin agonist treated animals showed a strong tendency to perform better over the entire experiment ((**c**), p = 0.061, one-way ANOVA/Tukey’s). During probe trials (time to first entry in the correct quadrant), the difference between high-GI and high-GI + ghrelin agonist were significant at tendency level only ((**b**), p = 0.096 for high GI vs. high GI + ghrelin agonist, p = 0.054 for high GI + ghrelin agonist vs. controls, one-way ANOVA/Tukey’s). Bars indicate SEM.

Among the three study groups (the group fed a high-GI diet, the group fed a high-GI plus ghrelin agonist, and the control group, which was fed an AIN-93G purified diet), the group fed a high-GI diet plus ghrelin agonist showed the best memory performance in the water maze (Fig. 1). Both in its learning dynamics in the course of the test days and in its performance in the probe trial, this group outperformed the other groups. However, the group fed a high-GI diet was not impaired in its cognitive performance compared with the control group as we originally hypothesized.

### Ghrelin agonist does not significantly affect Aβ plaque load or microglia activation

**Figure 2:**
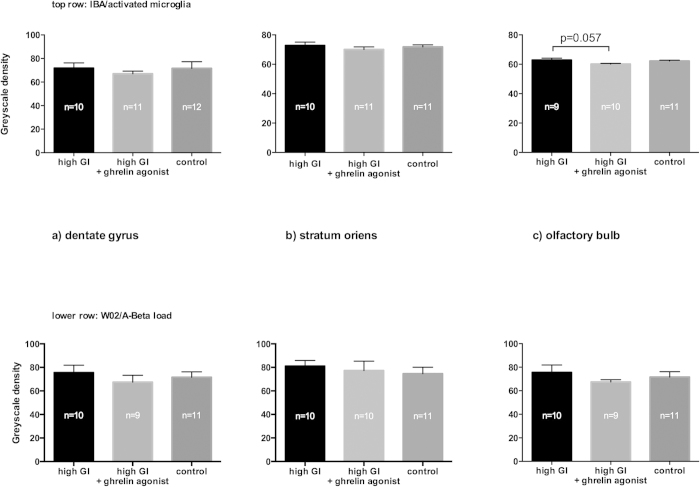
Neither markers for activated microglia (IBA, top row) nor for Aβ-load (W02, lower row) were significantly different after long-term ghrelin agonist treatment in the dentate gyrus (**a**) and stratum oriens (**b**). Only the level of activated microglia in the olfactory bulb of ghrelin-agonist-treated animals showed a tendency to be lower than in animals fed the high-GI diet alone ((**c**), Kruskal-Wallis test, followed by post-hoc Dunn’s multiple comparisons test, p = 0.057). Bars indicate SEM.

In a previous study we reported a positive influence of ghrelin on Alzheimer’s disease pathology markers such as Aβ plaque load (human Aβ4-10; see methods) and activated microglia^12^. In the current study, however, we did not observe any significant differences between the treatment groups in either of these immunohistochemical endpoints in the stratum oriens and dentate gyrus of the dorsal hippocampal area (Fig. 2(a,b)).

Because the olfactory epithelium has been shown to be involved at an early stage in Alzheimer’s disease^26^, we included the olfactory bulb in our immunohistochemical measurements. Microglia activation in the olfactory bulb was less in the group fed a high-GI diet plus ghrelin agonist than in the group fed a high-GI diet alone (p = 0.057, Fig. 2(c)). The Aβ plaque load in the olfactory bulb, however, did not differ significantly between these groups as measured in a grayscale density assessment (Fig. 2(c); see methods). Other research groups have reported an increased number of doublecortin (DCX)-positive cells after ghrelin treatment in the hippocampus of 2-month-old 5XFAD mice^27^. We did not observe any significant differences between groups in DCX-positive cell count in the dentate gyrus (data not shown).

### Long-term ghrelin agonist treatment leads to less weight gain, less overall food consumption, and more activity

**Figure 3:**
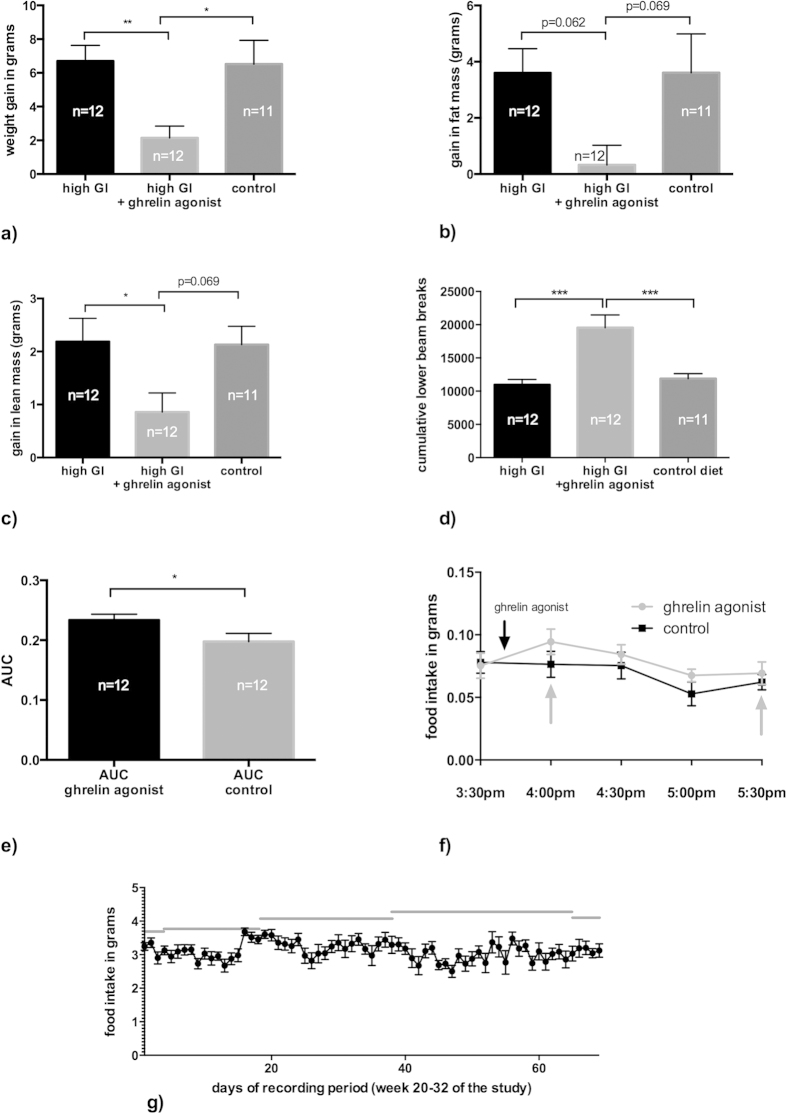
Over a period of 3 months ((**a**–**c**), compare timepoints “week 8” and “week 21” of the study), animals not treated with the ghrelin agonist gained significantly more weight than ghrelin agonist treated animals ((**a**), p = 0.009 for high-GI group vs. ghrelin agonist group, p = 0.015 for controls vs. ghrelin agonist group, one-way ANOVA/ Tukey’s). The same groups showed a tendency to gain more fat mass ((**b**), p = 0.062 for high GI vs. ghrelin agonist group, p = 0.069 for controls vs. ghrelin agonist group, one-way ANOVA/Tukey’s) than ghrelin agonist treated animals. The high-GI group gained significantly more lean mass than the ghrelin-agonist treated group ((**c**), p = 0.048), the controls showed a tendency ((c), p = 0.069, one-way ANOVA/Tukey’s). Activity levels during the mice’s active period (measurements taken in week 21) were higher in ghrelin-agonist-treated animals than in the high-GI and control diet groups ((**d**), p < 0.001 for both comparisons, one-way ANOVA/Tukey’s). Immediately after administration, the ghrelin agonist led to significantly higher food intake during the 2 subsequent hours ((**e**), p = 0.045 for AUC between gray arrows in (**f**), data for a sample of 12-month-old C57/BL6 mice from a different study, t-test for unpaired samples). However, cumulative food intake as measured for an entire day hardly ever reached the maximum of food assigned to ghrelin-agonist-treated animals as indicated by the gray lines ((**g**), days refer to the period while food intake was recorded). Bars indicate SEM.

Ghrelin and its agonists lead to overeating and obesity when food intake is unlimited^2^,^28^. Interestingly, the group fed a high-GI diet plus ghrelin agonist did not gain as much weight as did the other treatment groups (Fig. 3(a)). Only weight gain in the two groups not treated with the agonist was highly significant (Fig. 3(a), p = 0.009 for high-GI vs. high GI + ghrelin agonist group, p = 0.015 for controls vs. high-GI + ghrelin agonist group, ANOVA/Tukey’s). Of note, the increase in fat mass was particularly low in the group fed a high-GI diet plus ghrelin agonist (Fig. 3(b)). Because the food consumption of agonist-treated animals was limited to the average amount consumed by the group fed the high-GI diet alone (see methods), overeating triggered by the ghrelin agonist was not possible in this group. We observed a strong feeding response in our animals after the administration of the ghrelin agonist; however, the attempt to quantify this response in CLAMS metabolic cage measurements failed. The mice did not tolerate the procedure, mainly because of an accidentally shifted dark-night cycle. As a proof of concept, we have included CLAMS data from previous studies with C57/BL6 mice that clearly show the immediate feeding response after the administration of the same agonist LY444711 (Fig. 3(e,f)). Interestingly, daily recording of food intake in the group fed a high-GI diet plus ghrelin agonist over 8 weeks showed that the animals did not consume the full amount of food given to them daily (Fig. 3(g)). This finding and the overall elevated activity levels in agonist-treated animals compared with those fed the high-GI diet alone (p < 0.001 for high-GI/controls vs. ghrelin agonist treated group, ANOVA/Tukey’s, Fig. 3(d)) can explain the lesser weight gain in this treatment group.

### Long-term ghrelin agonist treatment does not impair glucose tolerance

**Figure 4:**
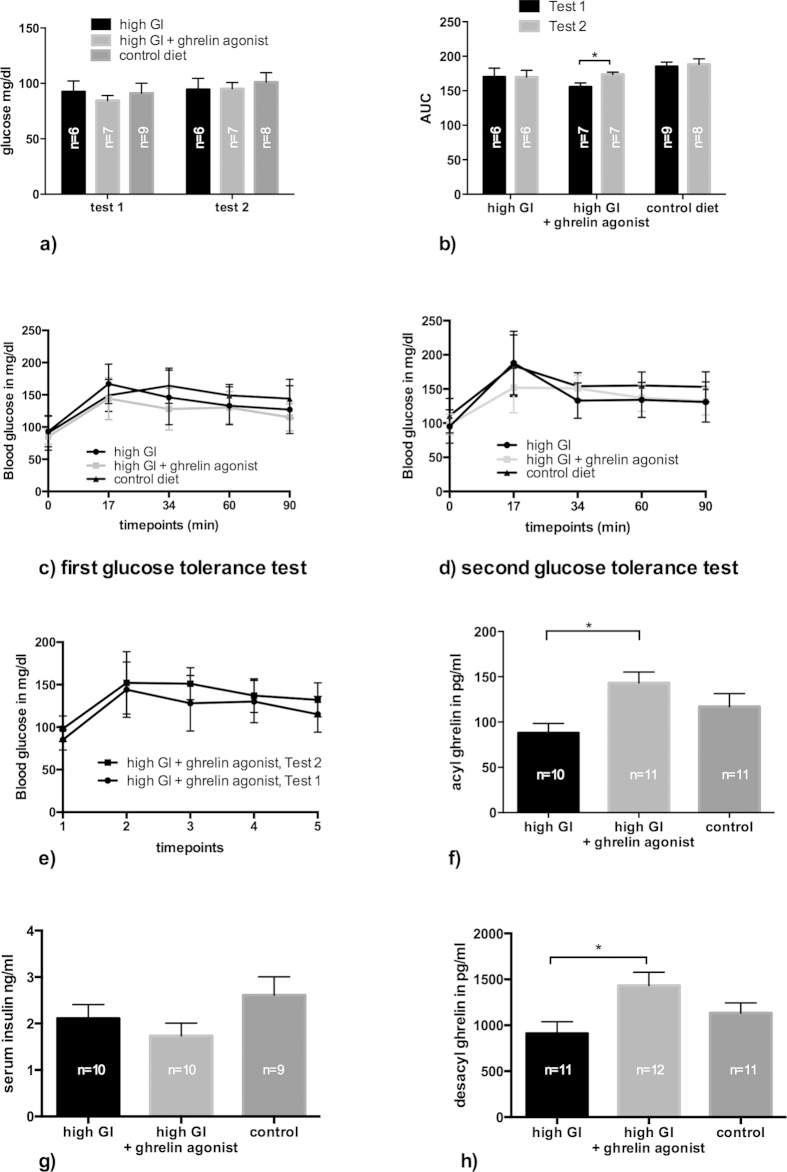
Baseline blood glucose levels after a six hours fasting period did not differ significantly between treatment groups ((**a**), one-way ANOVA/Tukey’s). Overall, the results of an oral glucose tolerance test were not influenced by long-term ghrelin agonist treatment (AUC = area under the curve, (**b**–**e**)). In the second test, animals from the high GI + ghrelin group were treated with the ghrelin agonist immediately before the glucose tolerance test and showed significantly higher blood glucose levels than in the first test (p = 0.010, t-test for paired samples, (**b**) and (**d**)). Both serum acyl ((**f**), p = 0.020, Kruskal-Wallis/Dunn’s) and desacyl ghrelin ((**h**), p = 0.020, ANOVA/Tukey’s) levels measured after a 6-hour fasting period were significantly higher in animals treated with the ghrelin agonist. There were no significant differences in serum insulin levels in the same samples ((**g**), one-way ANOVA/Tukey’s 4.7). Bars indicate SEM.

In order to characterize the impacts of a high-GI diet and long-term ghrelin agonist treatment on glucose metabolism, we performed an oral glucose tolerance test after 3 and 4 months of treatment. Baseline glucose levels after 6 hours of fasting (see methods) did not differ significantly between the groups in either test (Fig. 4(a)). A comparison of the area under the time curve (AUC) for both high-GI groups as well as the controls during the first test, which was performed shortly before the daily administration of the ghrelin agonist, did not reveal any differences. This suggests that neither the high-GI diet on its own nor in combination with long-term ghrelin agonist treatment impaired glucose tolerance (Fig. 4(b)). In order to clarify the ghrelin agonist’s short-term effects on glucose homeostasis, in the second glucose tolerance test, we treated animals with the ghrelin agonist immediately before administering the glucose load. In this experiment, the agonist-treated animals showed a significantly higher AUC than during the first test (p = 0.010, t-test for paired samples, Fig. 4(b)), whereas the mean AUC for the other groups did not change significantly. There were also no significant differences in the second test between groups. This result illustrates the differential effect of the ghrelin agonist on short-term and long-term glucose homeostasis.

We expected to see overall lower endogenous acyl ghrelin levels after long-term treatment with a ghrelin agonist, hypothesizing that artificially high ghrelin levels over a long period of time would lead to a down-regulation of endogenous production of the active peptide. However, both serum acyl and desacyl ghrelin levels as measured after a 6-hour fast were significantly higher in the group fed a high-GI diet plus ghrelin agonist than in the group fed the high-GI diet alone (Fig. 4(f,h)). A cross-reactivity in the assay between ghrelin agonist and endogenous ghrelin cannot be excluded with absolute certainty but appears both highly unlikely and probably insignificant because the last administration of the agonist took place 24 hours before the blood samples were taken. Insulin levels measured at the same time did not differ significantly between groups (Fig. 4(g)). It could be speculated that the long-term ghrelin agonist treatment led to a lower amount of ghrelin receptors in peripheral tissues, requiring higher circulating active ghrelin levels for the same metabolic effects. However, the differential analysis of peripheral tissues for endpoints relevant to insulin and ghrelin signaling was beyond the scope of this project. Possible future results of currently ongoing measurements will be discussed in a separate publication.

### Ghrelin agonist treatment beneficially influences central insulin signaling pathway

**Figure 5:**
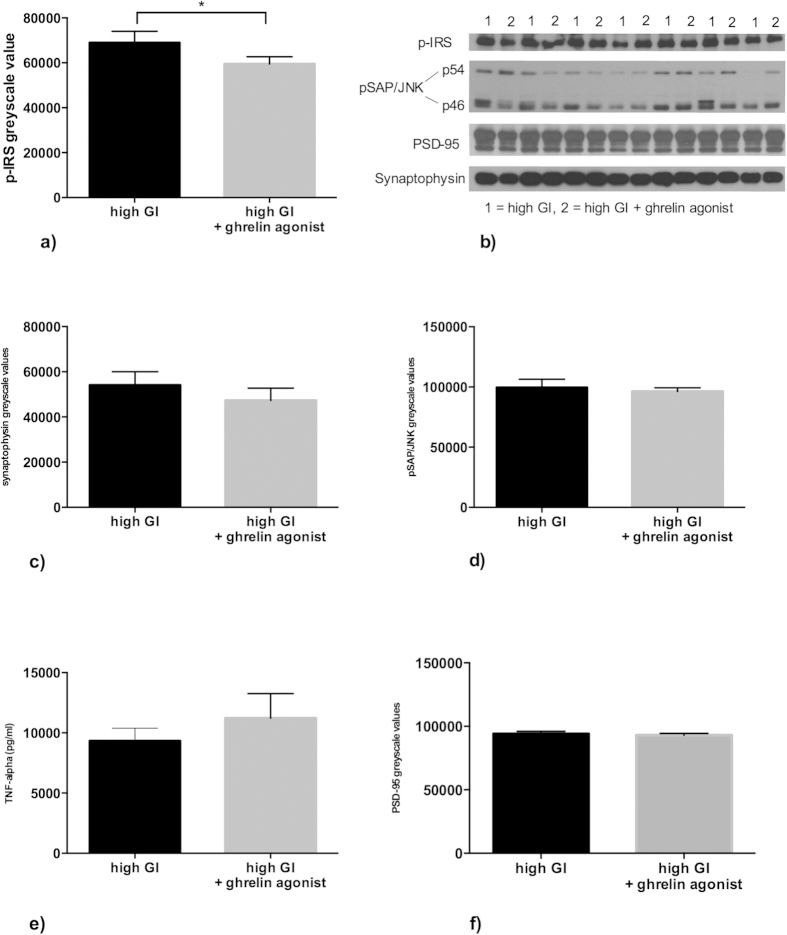
Animals treated with a ghrelin agonist showed a significantly lower amount of phosphorylated IRS (pIRS Ser636), which has been shown to be associated with impaired glucose tolerance ((**a**), p = 0.039, nonparametric Kolmogorov-Smirnov test). However, we did not detect any significant differences in hippocampal tissue between the high GI and high GI + ghrelin agonist groups for synaptophysin (**c**), pSAP/JNK (**d**), TNF-α (**e**) or PSD-95 (**f**). Bars indicate SEM.

Because other authors suggested an involvement of the tumor necrosis factor α (TNF-α)/c-Jun n-terminal kinase (JNK) pathway in Alzheimer’s disease triggered by Aβ-oligomers^29^, we measured TNF-α, pSAP-JNK, and phosphorylated insulin receptor substrate 1 (p-IRS Ser636) as well as synaptophysin and PSD-95 as synaptic markers in hippocampal brain tissue from the groups fed the high-GI diet (Fig. 5). We found a significant difference in p-IRS levels between the groups (Fig. 5(a), p = 0.039, nonparametric Kolmogorov-Smirnov test), indicating a possible interaction of long-term ghrelin agonist treatment with central insulin signaling. There was a moderate negative correlation in a linear regression analysis between behavioral results and p-IRS levels for both groups (r = −0.41); however, this correlation was not significant (p = 0.175, data not shown). We did not observe any differences in structural synaptic markers, neither presynaptically (synaptophysin) nor postsynaptically (PSD-95). Because there were no group differences in TNF-α or JNK-levels, we could not reproduce the TNF-α/JNK interrelations in Alzheimer’s disease in our mice.

## Discussion

Type 2 diabetes and Alzheimer’s type dementia are chronic diseases; consequently, all symptomatic treatments are intrinsically long-term. However, most studies of the interactions of ghrelin and insulin, which partly aimed to derive novel therapeutic pathways in diabetes, have looked at fairly short time frames of hours, days, or weeks^30^,^31^,^32^. In our study, we chose long-term ghrelin agonist administration in order to model the impacts of therapeutically influencing this system in a mammal over a period of several months. First, we could reproduce the previously known cognitive-enhancing effects of ghrelin and ghrelin agonists^14^, and at the same time we showed that this effect is seen even under the influence of a high-GI diet despite the ghrelin agonist’s short-term insulinostatic effect. The cognitive-enhancing effects were seen in the water maze test (Fig. 1), which is mainly a hippocampus-dependent spatial learning task^33^. This finding underlines the relevance of this ghrelin agonist’s cognitive effects in the Alzheimer’s type of dementia, which most prominently affects hippocampal brain areas and functions.

Most interestingly, we could show a long-term effect of ghrelin agonist treatment on metabolism that differed from its short-term actions on food consumption, weight development, and glucose tolerance. At the same time, we observed the well-known short-term orexigenic and insulinostatic effects of this endogenous peptide. These findings indicate a differential metabolic role of the ghrelin system in short-term and long-term treatment and call for a further differentiation of ghrelin’s long term role on glucose homeostasis, e.g. by including glucose clamp techniques in a long-term study design. Further, the observations in metabolic endpoints were made using the ghrelin agonist in combination with a high glycemic index diet. To what extent the results presented in this manuscript depend on this specific combination and to what extent they are also valid for a combination of a normal diet with a ghrelin agonist will be addressed in future and ongoing studies.

Given ghrelin’s differential interactions with insulin signaling, possibly also via mTORC1-dependent pathways^31^,^34^,^35^, we hypothesized a potentially protective effect of ghrelin agonist treatment on insulin signaling in the central nervous system. In agonist-treated animals, we found a lower expression of p-IRS-1 Ser636, which has been shown to be associated with both peripheral insulin resistance^36^, obesity^37^ and Alzheimer’s disease^29^. We therefore speculate that ghrelin and insulin signaling in the central nervous system are, to an extent, synergistic. On the one hand, the hormone reduces peripheral glucose uptake in periods of fasting, whereas on the other hand it improves or at least does not reduce glucose uptake in the central nervous system in situations of energy deficiency^38^.

A limitation of the interpretation of the present results is that the data are based on a mouse model for Alzheimer’s disease under the influence of a very specific high-GI diet. The latter might explain why we could not replicate the immunohistochemistry results of our previous study^12^. All extrapolation of these findings to other animal models must be done with care. Furthermore, we did not observe any structural differences in immunohistochemical markers for Aβ plaque load or central nervous system inflammation or in synaptic markers, which essentially leaves the task of identifying an immediate correlate of cognitive enhancement by ghrelin to future studies.

The present findings do suggest that any new therapeutic approaches in both diabetes and neurodegenerative diseases that are based on a manipulation of the ghrelin system must be addressed with utmost care. Counteracting ghrelin signaling for better glucose control or enhancing ghrelin signaling in the central nervous system for neuroprotection and cognitive enhancement are two tempting therapeutic pathways in neuroscience and endocrinology. However, both have to withstand long-term testing and the potentially contrasting effects of ghrelin and ghrelin agonists in peripheral tissues and in the brain.

## Methods

### Ethics statement

All animal protocols were approved by the University of Alabama at Birmingham Institutional Animal Care and Use Committee (IACUC). All methods were carried out in accordance with the approved guidelines and protocols.

### Animals, diets, and treatment

**Figure 6:**
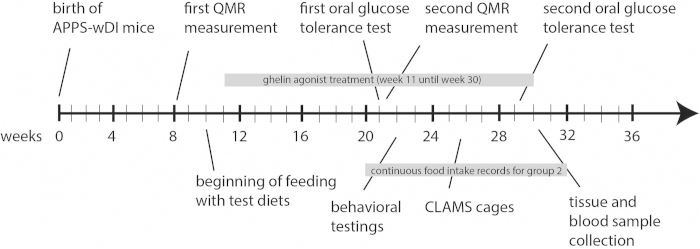
**Timeline of the study.**

The study timeline is shown in Fig. 6. A total of 36 male Tg APPSwDI (human APP with Swedish, Dutch, and Iowa mutations on a C57BL/6 background) were raised under equal dietary conditions for 2 months. At 10 weeks of age, the animals were divided into three groups of 12 animals each and received a diet consisting of 60% of kcal in carbohydrates with equal amounts of maltodextrin and sucrose plus either waxy maize starch (high-GI diet groups) or AIN-93G purified diet (controls). For detailed diet composition, see the supplementary material. During the first week of dietary acclimatization, all animals received a 45-mg sucrose pellet daily. After that, the group fed the high-GI diet plus ghrelin agonist received a 45-mg sucrose pellet containing 1.66% ghrelin agonist^39^ (LY444711; Eli Lilly, Indianapolis, IN) every day (30mg/kg/day, parallel to our previous study^12^, dose determined according to previous work by Giddings et al. 2008, abstract added to supplement); the other groups continued to be treated with sucrose pellets as placebos. Treatment took place daily at the same time between 2:00 and 4:00 pm during the animals’ light cycle and continued until the animals were sacrificed (treatment period: week 11 until week 30). Staff watched all animals take and eat the pellets and noted the days when the pellet was not consumed. This was only the case for few animals during dietary acclimatization. During the treatment period all animals ate the pellets. The amount of food consumed by all groups was measured every 2 weeks and the threshold of food restriction for the ghrelin-agonist-treated group was set at the average level of food consumption of the group fed the high-GI diet alone.

### Behavioral and cognitive assessments

All behavioral and cognitive assessments took place between weeks 22 and 24 (see Fig. 6). All tests took place during the light cycle. Feeding times were not changed throughout the assessments.

### Open field test

The maze consisted of a 42 by 42 cm^2^ arena with clear sides (20 cm high). The animal was placed in the arena and observed for 4 minutes with a camera-driven tracker system (Ethovision 9.5, Noldus, The Netherlands). The arena was subdivided into the open center area and the sides. The system recorded the position of the animal at 5 frames/s.

### Water maze

The water maze apparatus and procedure were described in detail before^40^. Briefly, we used a blue plastic pool, 120 cm in diameter, and a see-through round platform, 10 cm in diameter, located 0.5 cm below the water surface. During days 1 through 5 of the testing period, the mice were trained to find the hidden platform, which was kept in a constant position throughout these 5 days. Three trials were run per day; all starting positions were used equally in a pseudo-random order. The mice were given 60 s to find the platform and 10 s to stay on the platform. If the mouse did not find the platform in the assigned time, it was manually put onto the platform. The inter-trial interval during which the mouse was placed in a towel-bedded drying cage lasted 1 minute. Learning of the task was evaluated by recording the latency time to find the platform. At the end of the four trials on day 5 of the testing period, the mice were tested in a 60-s probe trial with no escape platform present. Mice that had learned the platform position predominantly searched in the “correct” quadrant of the pool during the probe trial or entered the correct quadrant faster. Trials were recorded by using a camera-driven tracker system (Ethovision 9.5, Noldus, The Netherlands).

### Zero maze

For the zero maze test, we used a round maze with a diameter of 61 cm designed for mice (SD Instruments, San Diego, CA). At the beginning of the trial, all mice were placed on the same open part facing in the same direction. Velocity, distances moved, and time spent in the open and closed parts were recorded for 4 minutes by using a camera-driven tracker system (Ethovision 9.5, Noldus, The Netherlands).

### Light-dark-box

We used a custom-built plastic light-dark box (46.5 cm length, 22 cm width, 28 × 22 cm light part, 18.5 × 22 cm dark part). Time spent in the light and dark parts as well as the number of entries into the dark part were recorded for 5 minutes by using a camera-driven tracker system (Ethovision 9.5, Noldus, The Netherlands). Mice were placed in the light part of the box facing away from the entrance to the dark part.

### Immunohistochemistry

Animals were sacrificed at week 30 for immunohistochemical, Western blot, and ELISA analyses. Mice were anesthetized with ketamine/xylazine (100/10 mg/kg) and perfused with cold saline. The brains were removed and cut in half sagittally, and the right hemisphere of the brain was placed in 4% paraformaldehyde overnight. The left hemisphere was dissected into four pieces (rostral cortex, caudal cortex, hippocampus, and midbrain/brainstem) and stored frozen at −80 ^o^C for protein analysis (ELISA, Western blot). The right half and the intact whole brains from 12 animals, 4 per group, were put in 30% sucrose for cryoprotection, and 30-μm thick coronal sections were cut on a freezing-sliding microtome.

Sections from 29 brains were stained for Aβ with the W0-2 antibody (human Aβ_4-10_; 1:2000; The Genetics Company, Schlieren, Switzerland). Another series of sections from the same 29 brains was stained for Iba-1 (1:1000; Wako, Richmond, VA) as a marker for activated microglia. For Aβ staining, sections were pretreated for 30 minutes in 85 °C sodium citrate solution (pH = 6.5). Following incubation with the primary antibody in TBS-T overnight at room temperature, tissues were rinsed three times and incubated with the appropriate biotinylated secondary antibody for 2 hours at room temperature. Sections were again rinsed three times and put for 2 hours with the tertiary antibody, extra Avidin-peroxidase. After another three rinses, metal-enhanced DAB staining was used for visualization. For each antibody, all sections were processed in one staining tray. All slides were air-dried, cleared in xylene, and coverslipped with DPX.

ImageJ software (NIH open source; http://imagej.nih.gov/ij/) was used to analyze the area occupied by Aβ and glial reactivity in stratum oriens of the dorsal hippocampus and in the dorsal dentate gyrus. Images of the appropriate brain areas were acquired with an Olympus DP70 digital camera. All images were acquired in one session to avoid changes in light levels. ImageJ measurements were performed by a scientist who was blind to the study design. Few images had to be excluded due to staining/tissue preparation problems (see Fig. 2).

### Oral glucose tolerance test

In order to avoid a priori differences in baseline blood glucose levels, mice had no access to food for a period of six hours before the glucose tolerance test. For the oral glucose tolerance test, 300 μl of a solution of 16.7 g glucose in 100 ml of purified water was administered directly into the mice’s stomach via gavage needles. Blood samples were taken from tail veins and immediately measured with the TRUE2Go blood measurement system^41^ for one baseline time point and then after 17, 34, 60, and 90 min. The mice were placed in a plastic retainer system during the procedure. One mouse was excluded from the analysis because it did not tolerate the gavage process.

### Protein extraction and Western blotting

For ELISA and Western blots, brain tissue was homogenized in RIPA (150 mM NaCl, 0.1% SDS, 0.5% sodium deoxycholate, 1% NP-40, 50 mM Tris, pH 8, 20 mM NaF, 2 mM EGTA, 0.5% levamisole, 1 mM NaVO4) plus protease inhibitor cocktail (p2714 Sigma-Aldrich, St Louis, MO) by use of the fast homogenization process Minilys® (Precellys, Bertin, France). After protein estimation with the Bradford method^42^, samples were diluted to an appropriate concentration.

For Western blotting, p-IRS Ser636 antibody (Santa Cruz Biotechnology, Dallas, TX), synaptophysin antibody clone SVP-38 (Sigma-Aldrich, St Louis, MO), pSAPK/JNK Thr183/Tyr185 (Cell Signaling Technologies, Danvers, MA) and PSD-95 antibody (Upstate/Millipore, Billerica, MA) were used. After electrophoresis and transfer to nitrocellulose, samples were incubated with the primary antibody overnight and were then incubated with the suitable secondary antibody for 90 minutes. For measuring TNF-alpha, a commercial ELISA kit was used (EMTNFA, ThermoScientific, Rockford, IL).

### Blood samples

Blood samples were taken after a 6-hour fasting period via intracardial puncture from the left ventricle shortly before the animals were perfused. Samples of 250 μl of blood were collected in chilled EDTA tubes (Becton, Dickinson and Company, Franklin Lakes, NJ) that were prefilled with 5 μl of 200 mM AEBSF stock yielding a final concentration of 4 mM AEBSF. Samples were centrifuged for 20 minutes at 17000 rpm and 4 °C and the plasma collected was immediately acidified with 200 μl of 1 M HCl per 1 ml of plasma. pH was adjusted accordingly before ELISA measurements for insulin and ghrelin. Acyl ghrelin and des-acyl ghrelin was measured with a custom-built 2-site sandwich ELISA^43^. For the measurement of insulin a commercially available ELISA kit was used (EZRMI-13K, EMD-Millipore, Billerica, MA).

### Quantitative magnetic resonance imaging

In vivo body composition (total body fat and lean tissue) of mice was determined by using an EchoMRI™ 3-in-1 quantitative magnetic resonance (QMR) machine (Echo Medical Systems, Houston, TX). A system test was performed by using a known fat standard before the measurements were taken. Mice were weighed and then placed into a clear holding tube capped with a stopper that restricted vertical movement but allowed constant airflow. The tube was inserted into the machine and the mouse was scanned by using Normal Precision mode.

### Metabolic cages

Twenty-four-hour patterns of food intake, energy expenditure (indirect calorimetry), and physical activity were measured by using CLAMS (Columbus Instruments Inc., Columbus, OH). This instrument also enforced the feeding regimens in an automated, computer-controlled manner. Body weight was monitored weekly.

### Activity measurements

Additional activity measurements over a period of five consecutive light and dark cycles were performed at week 21 by using a custom-built infrared-based beam-breaking system that recorded horizontal and vertical movements. Mice were placed in the system in their home cages with reduced bedding in order to not disrupt the continuous infrared measurements. Only data recorded on days 2 to 4 were included in the analysis.

### Statistical methods

All datasets were tested for Gaussian distribution using a D’Agostino & Pearson omnibus normality test. Whenever a normal (Gaussian) distribution could be validly assumed, a one-way ANOVA, then a post-hoc Tukey’s test for multiple comparisons was used to test for significant differences between groups (referred to as “ANOVA/Tukey’s”). Nonparametric samples were tested using the Kruskal-Wallis test and Dunn’s test for multiple comparisons as a post-hoc test (referred to as “Kruskal-Wallis/Dunn’s”). Whenever only two groups were involved in the measurements, differences were tested using a t-test for paired/unpaired samples in parametric distributions or a Kolmogorov-Smirnov test for nonparametric distributions. Being aware of the nested data problem^44^, we only compared values on the same level of analysis to decrease the likelihood of type-1 errors. All analyses were performed with GraphPad Prism software version 6.05 (GraphPad Software, Inc., La Jolla, CA).

## Additional Information

**How to cite this article**: Kunath, N. *et al.* Ghrelin agonist does not foster insulin resistance but improves cognition in an Alzheimer's disease mouse model. *Sci. Rep.* **5**, 11452; doi: 10.1038/srep11452 (2015).

## Supplementary Material

Supplementary Information
