# Supplementary material for: Ghrelin agonist does not foster insulin resistance but improves cognition in an Alzheimer’s disease mouse model
Source: Sci Rep. 2015 Jun 19;5:11452. doi: 10.1038/srep11452 (PMC4473679; doi:10.1038/srep11452)
Supplement: Supplementary Information [file srep11452-s1.pdf]

Supplementary material to:

Ghrelin agonist does not foster insulin resistance but improves cognition in an Alzheimer's disease mouse model

Nicolas Kunath(1) (3), Thomas van Groen (1), David B. Allison (2), Ashish Kumar (1), Monique Dozier-Sharpe (1), Inga Kadish (1)*

*correspondence and request for material should be addressed to "kunath@uab.edu"
(1) Department of Cell, Developmental and Integrative Biology, University of Alabama at Birmingham, Birmingham AL, USA
(2) Office of Energetics; Nutrition Obesity Research Center; Department of Nutrition Sciences, University of Alabama at Birmingham, Birmingham, AL, USA
(3) Department of Clinical Research, Max-Planck-Institute of Psychiatry, Munich, Germany

Content:

1. Diets (ingredients and composition)
2. Ghrelin/Sucrose pellets (ingredients and composition)
3. Abstract: Giddings, M., Cox, J., & Allison, D.B. (2008) "An Orally Available Ghrelin Agonist Chronically Increases Hunger in Mice"

1. Diets

1.1 Control Diet (AIN-93G Purified Diet)

**Formula**	**g/Kg**
Casein	**200.0**
L-Cystine	**3.0**
Corn Starch	**397.486**
Maltodextrin	**132.0**
Sucrose	**100.0**
Soybean Oil	**70.0**
Cellulose	**50.0**
Mineral Mix, AIN-93G-MX (94046)	**35.0**
Vitamin Mix, AIN-93-VX (94047)	**10.0**
Choline Bitartrate	**2.5**
TBHQ, antioxidant	**0.014**

NutrientInformation*

	**% by weight**	**% kcal from**
**Protein**	17.7	18.8
**Carbohydrate**	60.1	63.9
**Fat**	7.2	17.2
**Kcal/g**	**3.8**	

*Values are calculated from ingredient analysis or manufacturer data

Source: Harlan/Teklad custom research diet data sheet

1.2 High-GI diet (Fiber Adjusted Control Diet)

**Formula**	**g/Kg**
Casein	**200.0**
L-Cystine	**3.0**
AMIOCA (Waxy Maize Starch), customer supplied	**335.0**
Maltodextrin	**99.486**
Sucrose	**100.0**
Soybean Oil	**70.0**
Cellulose	**145.0**
Mineral Mix, AIN-93G-MX (94046)	**35.0**
Vitamin Mix, AIN-93-VX (94047)	**10.0**
Choline Bitartrate	**2.5**
TBHQ, antioxidant	**0.014**

Nutrient Information*

	**% by weight**	**% kcal from**
**Protein**	17.7	20.8
**Carbohydrate**	51.4	60.2
**Fat**	7.2	19.0
**Kcal/g**	**3.4**	

*Values are calculated from ingredient analysis or manufacturer data

Source: Harlan/Teklad custom research diet data sheet

2. Ghrelin/Sucrose pellets
2.1 Ghrelin pellet

Description

Modification of Sucrose Reward Tablet (see below), 1.66% Ghrelin (0.75 mg/45 mg tab).

Ingredients (%)

Sucrose Reward Tablet Mix (Sucrose, Microcrystaline Cellulose, Magnesium Stearate)	98.31
Ghrelin	1.66
Red Dye	0.03

Nutritional Profile

Fat, %		0.0
Protein, %		0.0
Minerals, %		
	Calcium	0.02
	Phosphorus	0.01
	Magnesium	0.04
	Chloride	0.04
	Others	0.00
Fiber (max), %		4.6
Carbohydrates, %		91.6
Energy (kcal/g)		3.8
	From Carbohydrates	3.8
	From Fat	0
	From Protein	0

2.2 Sucrose pellet

Description

Sucrose Reward Tablet (precision pellet)

Ingredients (%)

Sucrose, Dextrose, Magnesium Stearate, Inert Binder

Nutritional Profile

Fat, %		0.0
Protein, %		0.0
Minerals, %		
	Calcium	0.02
	Phosphorus	0.01
	Magnesium	0.04
	Chloride	0.04
	Others	0.00
Fiber (max), %		4.7
Nitrogen-free extract (by difference), %		85.1
	Glucose	58.97
	Fructose	30.00
	Sucrose	64.37
Energy (kcal/g)		3.4
	From Carbohydrates	3.4
	From Fat	0
	From Protein	0

3. Giddings, M., Cox, J., & Allison, D.B. (2008) “An Orally Available Ghrelin Agonist Chronically Increases Hunger in Mice” (210-P Presentation to 2008 The Obesity Society Annual meeting, in Phoenix, AZ, October 3–7, 2008. Abstract published in Obesity, 16(S1): S108)

An Orally Available Ghrelin Agonist Chronically Increases Hunger in Mice

Matthew Giddings, James Cox, David Allison Birmingham, AL

Caloric restriction (CR) has been found to extend longevity in a wide range of species. According to the principle of hormesis, this action may stem, at least in part, from the moderate stress resulting from a state of chronic hunger. Here we report preliminary results with a potential CR mimetic, LYY444711 (Lilly), a ghrelin receptor agonist. Previous observations have suggested that the gut peptide ghrelin acts as an orexigenic signal mediating psychological effects of caloric restriction (CR). In the current study, we found that motivation for food, as reflected in an operant conditioning model, is increased by this drug with no sign of tachyphylaxis across 8 weeks of treatment. Adult, male C57BL/6J mice were trained to lever press for 20 mg pellets of their maintenance diet, AIN-93M. One group of mice (N=6) received a daily oral dose of LY444711 (30 mg/kg) dissolved in 100 mg chocolate pills for 8 weeks, with their food intake maintained at the mean intake of the vehicle control group (N=6). Progressive ratio responding was assessed in terms of total responses, breakpoint ratio, and number of reinforcements at four time points: immediately prior to start of treatment (baseline) and after 3, 5, and 8 weeks. Performance by the groups during the treatment period was compared by analysis of covariance with baseline responding as the covariate. The treatment group showed significantly greater progressive ratio responding on all three measures as indicate by significant Group effects (P values < .05). The stability of the effect of LY444711 over time was indicated by the lack of a significant Group x Replication interaction for any of the response measures (P values > .25). Thus, these results suggest the efficacy of LY444711 in maintaining a state of chronic hunger in mice. Longterm treatment with LY444711 could provide a model to test the hypothesis that hunger-induced hormetic effects of CR contribute to its anti-aging effects.
*M.G. was supported by a predoctoral training grant from Kraft Foods, Inc. We thank Eli Lilly & Company for graciously supplying LY444711.
